# MiR-23a-depressed autophagy is a participant in PUVA- and UVB-induced premature senescence

**DOI:** 10.18632/oncotarget.9357

**Published:** 2016-05-13

**Authors:** Jia-an Zhang, Bing-rong Zhou, Yang Xu, Xu Chen, Juan Liu, Maya Gozali, Di Wu, Zhi-qiang Yin, Dan Luo

**Affiliations:** ^1^ Department of Dermatology, The First Affiliated Hospital of Nanjing Medical University, Nanjing, Jiangsu, China; ^2^ Jiangsu Provincial Key Laboratory of Molecular Biology for Skin Diseases and STIs, Nanjing, Jiangsu, China; ^3^ Institute of Dermatology, Chinese Academy of Medical Sciences (CAMS) & Peking Union Medical College (PUMC), Nanjing, Jiangsu, China

**Keywords:** autophagy, stress-induced premature senescence, miR-23a, AMBRA1, ultraviolet, Gerotarget

## Abstract

Autophagy is a cellular catabolic mechanism that is activated in response to stress conditions, including ultraviolet (UV) irradiation, starvation, and misfolded protein accumulation. Abnormalities in autophagy are associated with several pathologies, including aging and cancer. Furthermore, recent studies have demonstrated that microRNAs (miRNAs) are potent modulators of the autophagy pathway. As a result, the current study aims to elucidate the role of the autophagy-related miRNA miR-23ain the process of photoaging. Experiments demonstrated that the antagomir-mediated inactivation of miR-23a resulted in the stimulation of PUVA- and UVB-depressed autophagy flux and protected human fibroblasts from premature senescence. Furthermore, AMBRA1 was identified as a miR-23a target. AMBRA1 cellular levels increased following the introduction of miR-23a antagomirs. And a bioinformatics analysis revealed that the AMBRA1 3′ UTR contains functional miR-23a responsive sequences. Finally, it was also demonstrated that both AMBRA1 overexpression and Rapamycin treatment were both able to rescue fibroblasts from PUVA and UVB irradiation-induced autophagy inhibition, but that these effects could also be mitigated by miR-23a overexpression. Therefore, this study concludes that miR-23a-regulated autophagy is a novel and important regulator of ultraviolet-induced premature senescence and AMBRA1 is a rate-limiting miRNA target in this pathway.

## INTRODUCTION

Chronic exposure to solar ultraviolet radiation (UV) can induce photoaging [1]. *Ex vivo* experiments have shown that the repeated exposures of human skin fibroblasts to UVB or 8-methoxypsoralen plus ultraviolet-A irradiation (PUVA) at subcytotoxic levels triggers ultraviolet stress-induced premature senescence (SIPS) [2, 3]. Under these conditions, fibroblasts cease to divide, and instead undergo a series of dramatic morphological and metabolic changes [4]. *In vitro* studies have demonstrated that cell senescence can occur due to a variety of processes including genetically programmed pathways, telomere shortening, and the accumulation of DNA damage [5].

Autophagy, the dynamic process of degrading unnecessary or dysfunctional cell components, has also been linked to aging [6]. Studies have shown that reduction in autophagy can accelerate the aging process, while the stimulation of autophagy may have potent anti-aging effects [7, 8]. However, the role of autophagy specifically in photoaging has not been thoroughly studied. And the underlying molecular mechanism linking autophagy to photoaging is still not known.

Furthermore, miRNAs have also been linked to the process of aging and senescence. MiRNAs are endogenously expressed small RNA molecules that mediate posttranscriptional gene silencing and have the capacity to simultaneously regulate tens to hundreds of target genes [9]. As a result, they are potential targets for anti-aging, and more specifically anti-photoaging, therapy [10, 11]. For example, a recent unbiased miRNA screen discovered that miR-34c-5p human primary dermal fibroblasts from UVB-induced premature senescence *via* regulations of some senescence-related molecules [12]. Furthermore, the recent experiments demonstrated that miR-23a-3p was up-regulated in both aged and senescent fibroblasts and miR-23a expression was remarkably up-regulated in HaCaT cells after the UVB irradiation [13, 14].

Furthermore, miRNAs have also been shown to regulate autophagy pathways. While autophagic activity is regulated by a variety of factors, including insulin receptor-signaling pathway, the TOR pathway, Sirt1, and caloric restriction [15]. Several miRNAs, such as miR-30a, miR-101, miR-130a, and miR-196, have also been implicated [16]. While the role of miRNAs in autophagy has been established, and the role of autophagy in aging, it has not yet been demonstrated whether miRNAs have any role in photoaging.

However, miR-23a serves as a promising target as the link between miRNA expression and photoaging, as it has been reported to be up-regulated in several *in vitro* and *in vivo* aging models [17–19]. But how miR-23a-mediated autophagy mediates the development of ultraviolet stress-induced premature senescence has yet to be established. Therefore, the aim of the current study is to identify this role. In order to do so, two stress-induced premature senescence models were created by repeated subcytotoxic exposures of dermal human fibroblasts to either UVB or PUVA irradiation. The relation between miR-23a expression levels and autophagy levels in both PUVA- and UVB-SIPS fibroblasts was then evaluated. Furthermore, the molecular target of miR-23a was also identified *via* a bioinformatics approach in an effort to elucidate the mechanism of regulation of miR-23a.

## RESULTS

### Decreased autophagy flux in PUVA- and UVB-SIPS fibroblasts

**Figure 1:**
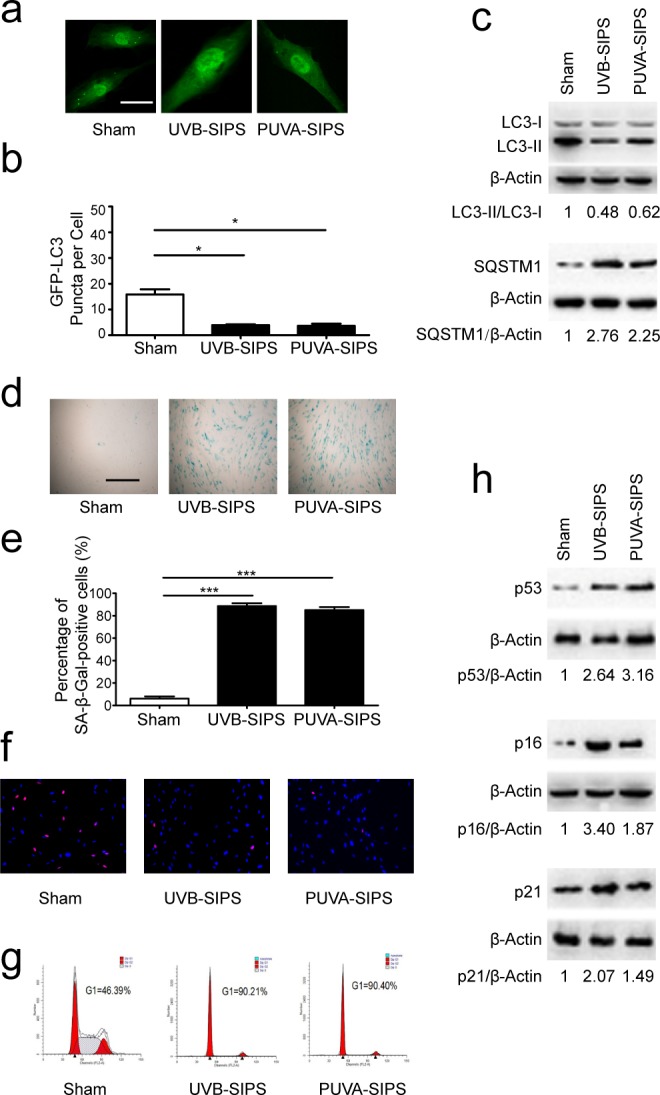
Autophagy is down-regulated in PUVA-SIPS and UVB-SIPS fibroblasts **a.** Cells were transiently transfected with GFP-LC3, and then treated with 10 J/cm^2^ of PUVA for 14 days or 25mJ/cm^2^ of UVB twice a day for 5 days to establish PUVA- and UVB-SIPS models. Representative images were taken by confocal microscopy. Scale bars = 50μm. **b.** The percentage of cells with greater than 10 GFP-LC3 puncta was counted on the images. (means ± SEM of the independent experiments, *n* = 3, *p < 0.05). **c.**, **h.** Cells were collected for western-blotting analysis using LC3-, p62-, p53, p16 or p21-specific antibodies. Actin was used as a loading control. The LC3-II/LC3-I, SQSTM1/Actin, p53/Actin, p16/Actin, and p21/Actin densitometric ratios were marked (3 independent experiments gave similar results. See Figure S1). **d.** SA-β-Gal staining was performed. Scale bars = 100μm. **e.** The cellular senescence was determined by SA-β-gal staining. Premature senescence cells stained blue. The percentage of SA-β-Gal positive cells was calculated. (means ±SEM of the independent experiments, *n* = 3, ***p < 0.001). **f.** The cells were stained by EdU and Hoechst two days post UV-irradiation. **g.** G1 arrest was analyzed by flow cytometry.

Confocal microscopy revealed that PUVA and UVB irradiation could repress GFP-LC3 puncta formation in fibroblasts, indicating that autophagy is inhibited under these conditions (Figure 1a-1b). The lipid conjugation of free LC3-I to the autophagic membrane-associated LC3-II was attenuated in the extracts of the cells following subcytotoxic ultraviolet irradiation, and the degradation of the autophagic cargo receptor protein p62/SQSTM1 was reduced in sham-irradiated cell extracts (Figure 1c and Figure S1). We confirmed that autophagy was down-regulated in PUVA-SIPS and UVB-SIPS fibroblasts. We also demonstrated increases in senescence-related expressions of SA-β-gal, p16, p53, and p21, as well as an increase in G1 cell cycle arrest and a decrease in the percentage of EdU-positive cells in the PUVA-SIPS and UVB-SIPS fibroblasts (Figure 1d-1h and Figure S1) [20].

### Expression and effects of miR-23a ~27a ~24-2 cluster in PUVA- and UVB-SIPS fibroblasts

**Figure 2:**
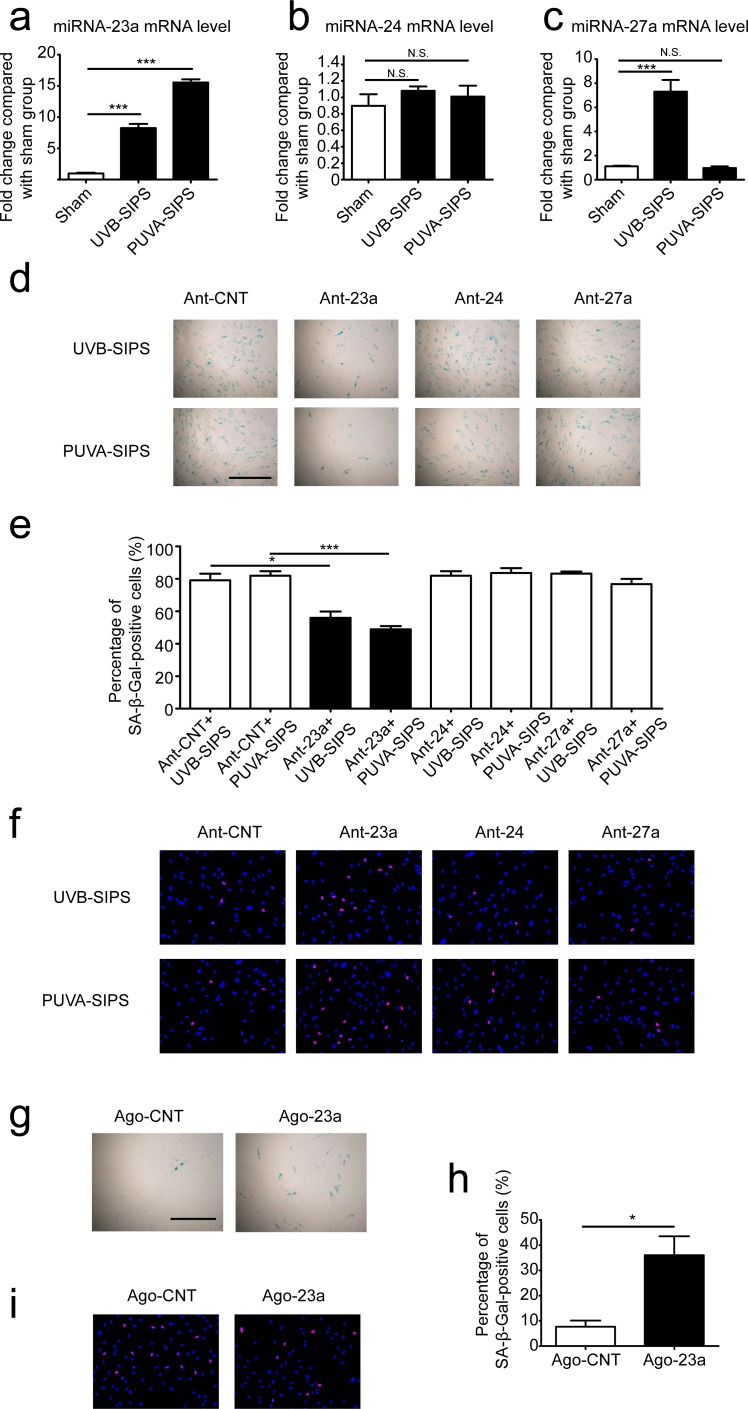
The roles of miR-23a on the SA-Δ-gal-positive percentages in PUVA-SIPS and UVB-SIPS fibroblasts **a.**, **b.**, **c.** The expression levels of miR-23a, miR-27a and miR-24 were detected *via* qRT-PCR in the UVB- and PUVA-SIPS fibroblasts as well as the sham-irradiated cells groups. (means ±SEM of the independent experiments, ***p < 0.001, N.S., not significant). **d.** Prior to ultraviolet irradiation, cultured fibroblasts were transfected with miR-23a antagomirs (Ant-23a) or miR-24 antagomirs (Ant-24) in addition to either miR-27a antagomirs (Ant-27a) or control antagomirs (Ant-CNT). The transfected fibroblasts were irradiated to establish the UVB- and PUVA-SIPS fibroblasts. SA-Δ-gal staining was then performed. **e.** The percentage of SA-Δ-gal-positive cells were calculated (means ±SEM of the independent experiments, ***p < 0.001, *p < 0.05). **f.** The percentage of EdU-positive cells was shown. **g.** Cultured fibroblasts were transfected with miR-23a agomir (Ago-23a) or control agomir (Ago-CNT). SA-β-Gal staining was performed. Scale bars = 100μm. **h.** The percentage of SA-Δ-gal-positive cells were calculated (means ±SEM of the independent experiments, *p < 0.05). **i.** The percentage of EdU-positive cells was shown.

The “miR-23a cluster” (miR-23a, miR-27a, miR-24-2) is located on human chromosome 19p13.2, and is expressed from its own upstream promoter, located in the 2600 to +36 bp region, which includes a GC-rich region and a transcription start site (0 to 124 bp). The expression level of miR-23a was increased in PUVA- and UVB-SIPS fibroblasts. Meanwhile, miR-24-2 showed no obvious changes in PUVA- and UVB-SIPS fibroblasts, while miR-27a expression was up-regulated only in UVB-SIPS fibroblasts (Figure 2a-2c). To detect the effects of endogenous miRNA inhibition on SIPS, we transfected PUVA- and UVB-SIPS cells with miR-23a-specific antagomirs (Ant-23a), miR-24-specific antagomirs (Ant-24), miR-27a-specific antagomirs (Ant-27a), and Ant-CNT (Ant-CNT), and then analyzed the percentage of SA-Δ-gal-positive and EdU-positive cells. Antagomirs such as the ones used in these experiments are anti-mRNA molecules that inhibit endogenous mature miRNAs. We demonstrated that the under-expression of miR-23a decreased SA-Δ-gal-positive cells and increase EdU-positive cells in PUVA- and UVB-SIPS fibroblasts. However, the same effect was not found in the miR-27a and miR-24 intervention groups (Figure 2d-2f). Furthermore, fibroblasts were transfected with miR-23a-specific agomirs (Ago-23a, a cholesterylated miRNA mimic shown to have similar *in vitro* effects as endogenous miRNA) and then analyzed the percentage of SA-Δ-gal-positive and EdU-positive cells (Wang XG, et al., 2013). These results demonstrate that over-expression of miR-23a increased SA-Δ-gal-positive cells and decreased EdU-positive cells in non-ultraviolet irradiation group (Figure 2g-2i).

### MiR-23a-specific antagomirs (Ant-23a) stimulated autophagy in PUVA- and UVB-SIPS fibroblasts

**Figure 3:**
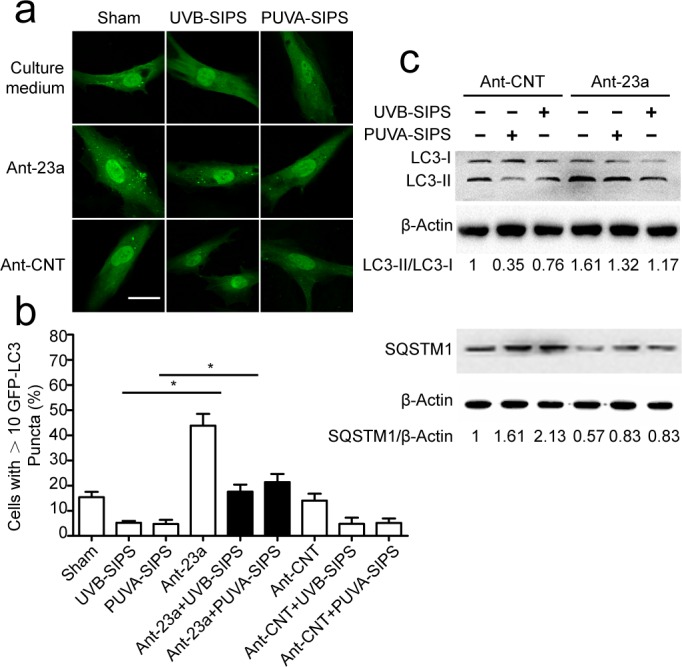
The down-expression of miR-23a resulted in increased autophagic activity in the PUVA- and UVB-SIPS fibroblasts Fibroblasts were transfected with Ant-23a or Ant-CNT and evaluated for autophagic levels. **a.** Representative confocal images of GFP-LC3 in fibroblasts before or after UVB- or PUVA-SIPS. Scale bars = 50μm. **b.** The percentage of cells having induced autophagy was determined as the percentage of GFP-LC3 cells with greater than 10 GFP-LC3 puncta per cell (means ±SEM of the independent experiments, *p < 0.05). **c.** Ant-23a reversed the UVB- or PUVA-SIPS-induced decrease of LC3-I to LC3-II conversion and decrease of SQSTM1/p62 in the fibroblasts (*n* = 3). Actin was used as a loading control. The LC3-II/LC3-I and SQSTM1/Actin ratios are marked (3 independent experiments gave similar results. See Figure S2).

To investigate the effects of endogenous miR-23a inhibition on autophagy, fibroblasts were transfected with either miR-23a -specific antagomirs (Ant-23a) or control antagomirs (Ant-CNT) and analyzed for autophagic activity. As shown in Figure 3a and 3b, after ultraviolet irradiation, down-expression of miR-23a significantly increased GFP-LC3 dot formation. Meanwhile, lipid conjugation of free LC3-I to the autophagic membrane-associated LC3-II was stimulated following Ant-23a transfection (Figure 3c and Figure S2). Hence, autophagy was accelerated in UV-SIPS cells transfected with Ant-23a, but not with control antagomirs. Moreover, SQSTM1 degradation was more prominent after Ant-23a transfection compared with Ant-CNT groups (Figure 3c and Figure S2). Therefore, inhibition of endogenous miR-23a using antagomirs led to a further stimulation of the autophagic activity in UV-SIPS cells, suggesting that endogenous miR-23a contributes to the limitation of stress-activated autophagic cell responses.

### MiR-23a-specific antagomirs (Ant-23a) suppressed senescence in PUVA- and UVB-SIPS fibroblasts

**Figure 4:**
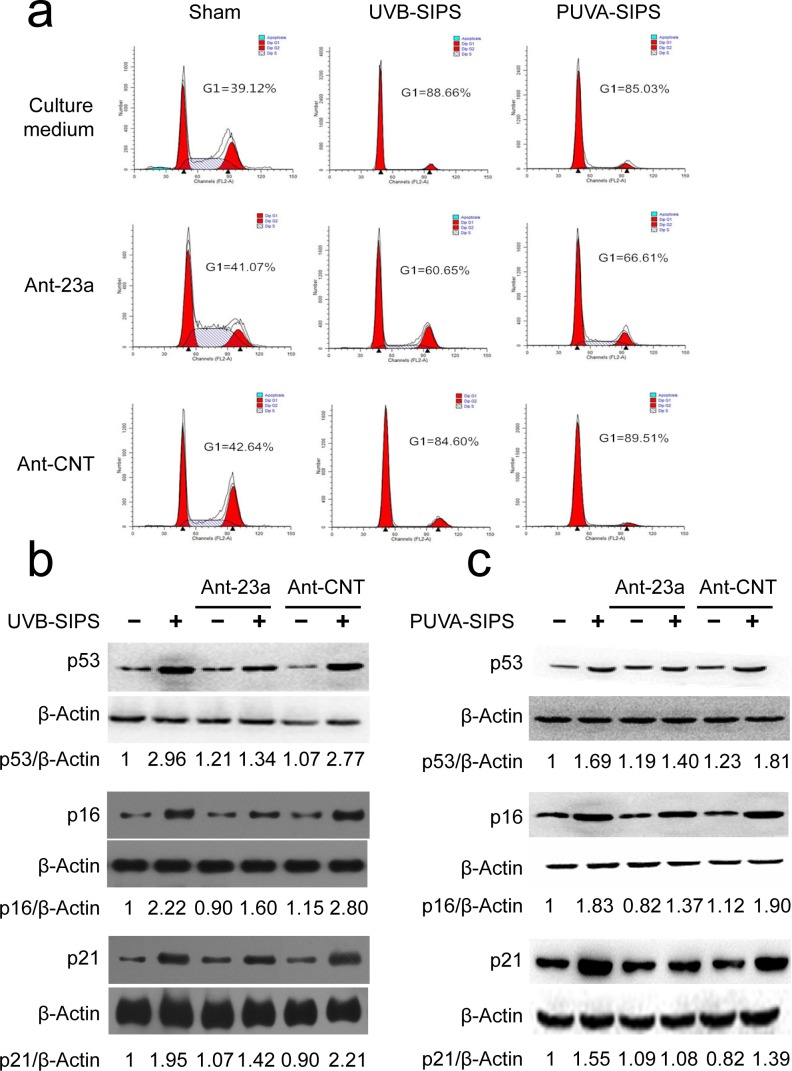
miR-23a downexpression suppressed the PUVA- and UVB-induced SIPS **a.** The G1 arrest was examined in the Ant-23a- or Ant-CNT-transfected cells with or without UV irradiation. **b.**, **c.** The relative protein levels of p53, p16, and p21 were examined in the Ant-23a- or Ant-CNT-transfected cells with or without UV irradiation (*n* = 3). Actin was used as a loading control. The p53/Actin, p16/Actin, and p21/Actin densitometric ratios were marked (3 independent experiments gave similar results. See Figure S3).

The above results demonstrated that under-expression of miR-23a may decrease SA-ß-gal-positive cells and increase EdU-positive cells in PUVA- and UVB-SIPS fibroblasts, suggesting an anti-SIPS effect following Ant-23a transfection. To further confirm these results, experiments were performed to characterize the effect of miR-23a underexpression on G1 arrest and p53, p16, and p21 protein expression. The UV-irradiated cells transfected with Ant-23a showed a significant decrease in G1 phase cell proportions compared to those UV-irradiated cells transfected with Ant-CNT (Figure 4a). Similar changes could be found in the levels of p53, p16, and p21 proteins (Figure 4b-4c and Figure S3). The data show that under-expression of miR-23a in human dermal fibroblasts can delay PUVA- and UVB-induced premature senescence.

### MiR-23a inhibits AMBRA1 expression by targeting its 3′-UTR

**Figure 5:**
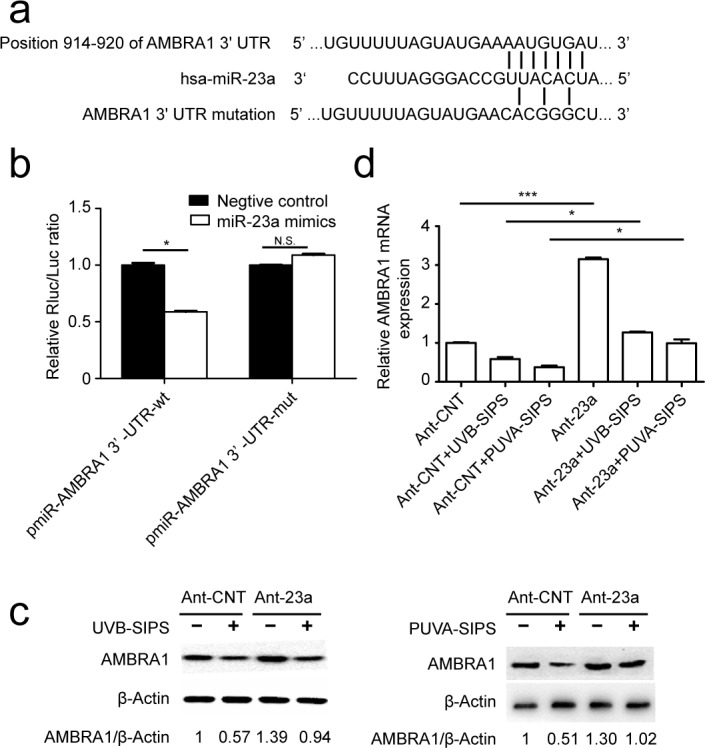
AMBRA1 as a direct target of miR-23a- and miR-23aaffected AMBRA1 levels in fibroblasts **a.** The “seed region” of the miR-23a in the AMBRA1-3′-UTR-wt. For the pmiR-AMBRA1-3′-UTR-mut, 7 nucleotides AAUGUGA were changed to CACGGGC. wt, wild type; mut, mutation. **b.** Analysis of luciferase activity. Luciferase assays were done on293T cells. Renilla luciferase was used as an internal control (means ±SEM of the independent experiments, *p < 0.05, N.S. not significant). **c.** Protein levels by western blot (3 independent experiments gave similar results. See Figure S5) and mRNA expression levels(means ±SEM of the independent experiments, *p < 0.05***p < 0.001, *p < 0.05) by qRT-PCR. **d.** of AMBRA1 show up-regulation of expression levels in fibroblasts transfected with Ant-23a compared with Ant-CNT.

AMBRA1 (GenBank accession number: NM_017749) was identified as a miR-23a target using the following bioinformatics methods. The predicted interaction between miR-23a and the AMBRA1 3′ UTR is shown in Figure 5a. To confirm whether AMBRA1 is a direct target of miR-23a in fibroblasts, we cloned either a 1038-bp fragment of the AMBRA1 3′-UTR containing the target sequence or a fragment of the 3′-UTR containing a target site mutated to be a luciferase reporter vector (Figure S4) and investigated the effect of miR-23a on the luciferase activity of each construct in 293T cells. As shown in Figure 5b, miR-23a suppressed the luciferase activity of the pmiR-AMBRA1-wt compared with the negative control, while the mutation of the miR-23a binding site blocked this suppressive effect. We further performed an immunoblot analysis in control and Ant-23a-transfected cell extracts using an AMBRA1-specific antibody. AMBRA1 protein levels were increased when miR-23a was underexpressed in PUVA- and UVB-SIPS fibroblasts (Figure 5c and Figure S5). Similarly, the introduction of the Ant-23a but not Ant-CNT resulted in an increase in AMBRA1 mRNA levels in the PUVA- and UVB-SIPS fibroblasts (Figure 5d).

### miR-23a mitigated rapamycin-induced autophagy and anti-senescence in PUVA- and UVB-SIPS fibroblasts

**Figure 6:**
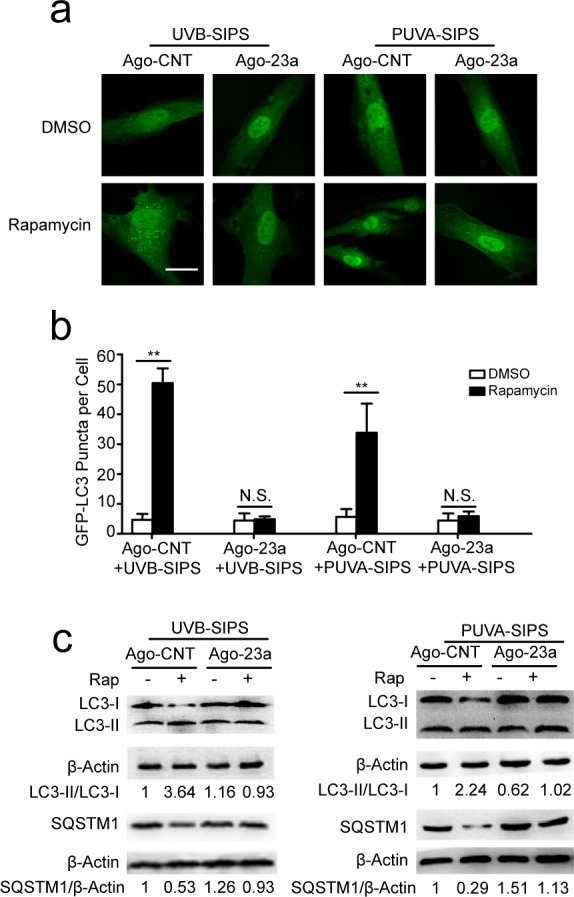
Overexpression of miR-23a blocked rapamycin-induced increased autophagy activity **a.** Prior to UVB or UVA irradiation, cells were co-transfected with Ago-23a or control (Ago-CNT) and GFP-LC3 adenovirus, GFP-LC3 dot formation was assessed following DMSO (carrier) or rapamycin(10 nM, 24h). **b.** Quantitative analysis of GFP-LC3 positive cells in (a) (means ±SEM of the independent experiments, *p < 0.05, **p < 0.01, N.S., not significant). Scale bars = 50μm. **c.** Ago-23a reversed the rapamycin-induced increase of LC3-I to LC3-II conversion and decrease of SQSTM1 in the fibroblasts. Actin was used as a loading control. The LC3-II/LC3-I and SQSTM1/Actin, densitometric ratios were marked (3 independent experiments gave similar results. See Figure S6).

**Figure 7:**
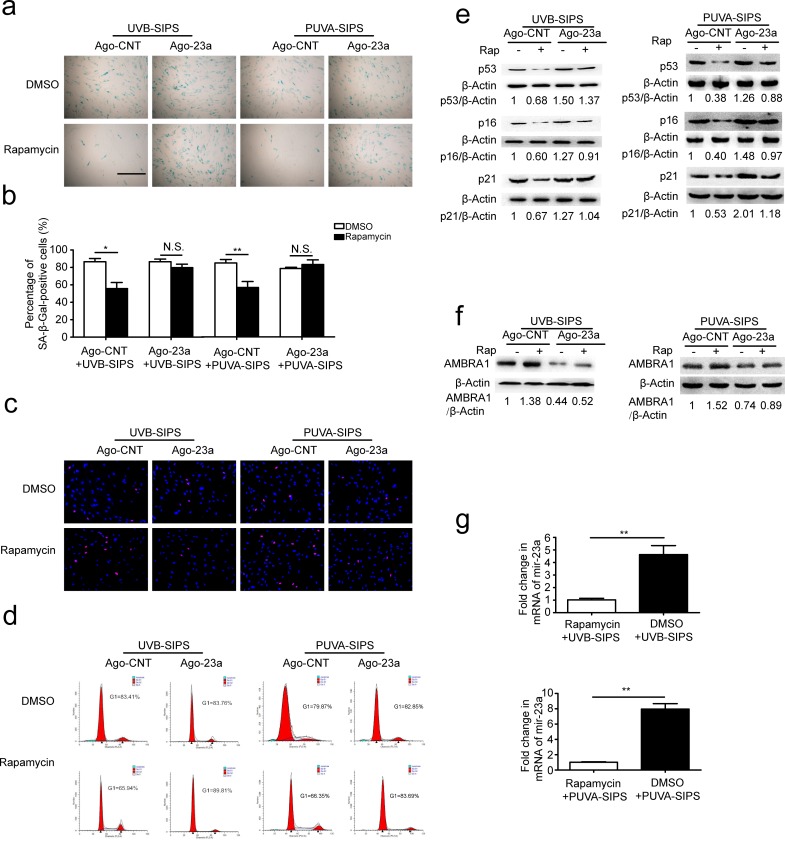
Overexpression of miR-23a blocked rapamycin-induced anti-SIPS activity **a.** Prior to UV irradiation, cells were co-transfected with Ago-23a or control (Ago-CNT) and GFP-LC3 adenovirus. SA-ß-gal staining was performed. Scale bars = 100μm. **b.** The percentage of SA-ß-gal-positive cells were calculated (means ±SEM of the independent experiments, *p < 0.05, N.S., not significant). **c.** The cells were stained by EdU and Hoechst two days post UV irradiation. The percentage of EdU-positive cells was shown **d.** G1 arrest was examined. **e.** The relative protein levels of p53, p16, and p21 were examined. Actin was used as a loading control. The p53/Actin, p16/Actin and p21/Actin, densitometric ratios were marked (3 independent experiments gave similar results. See Figure S7). **f.** Prior to UV irradiation, Immunoblots of Ago-23a or control (Ago-CNT) transfected cells that were treated with DMSO or rapamycin(10 nM, 24h) (3 independent experiments gave similar results. See Figure S7). **g.** qRT-PCR analysis of miR-23a mRNA levels(means ±SEM of the independent experiments, **p < 0.01).

Prior to ultraviolet irradiation, fibroblasts were treated with 10 nM Rapamycin, an autophagy agonist. Our results suggest that Rapamycin can increase autophagy, as evidenced by increased accumulation of GFP-LC3 puncta and LC3-II accumulation, and decreased SQSTM1/p62 level in PUVA- and UVB-SIPS fibroblasts transfected by Ago-CNT. Moreover, overexpression of miR-23a led to the attenuation of GFP-LC3 puncta formation (Figure 6a-6b), decrease in protein levels of LC3 II, and prevention of SQSTM1/p62 degradation (Figure 6c and Figure S6), confirming the inhibitory effect of this miRNA on autophagy. AMBRA1 protein level was increased in Rapamycin-treated cells (Figure 7f and Figure S7), and endogenous miR-23a level was decreased following rapamycin treatment (Figure 7g). Furthermore, Rapamycin increased EdU-positive cells and decreased SA-β-gal positive cell percentages and G1 phase-arrested cell percentages, as well as p16, p53, and p21 protein levels in PUVA- and UVB- SIPS fibroblasts transfected by Ago-CNT. However, these effects were suppressed in fibroblasts transfected by miR-23a agomirs (Figure 7a-7e and Figure S7). These results suggest that rapamycin-induced autophagy rescues fibroblasts from ultraviolet-induced premature senescence and that this pathway is negatively regulated in part by miR-23a.

### Upregulation of AMBRA1 activated autophagic flux and restrained senescence in PUVA- and UVB-SIPS fibroblasts and its effects can be mitigated by miR-23a agomirs

**Figure 8:**
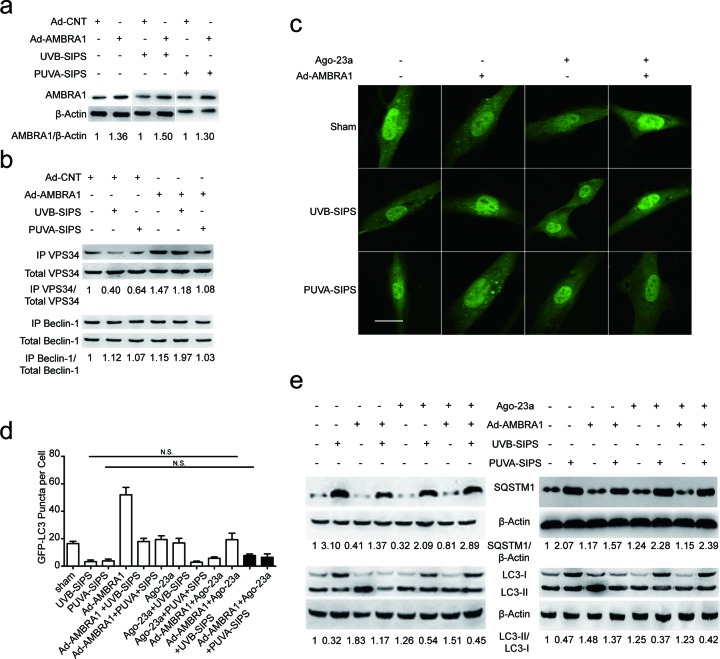
Overexpression of miR-23a blocked Ad-AMBRA1-induced increased autophagy activity **a.** AMBRA1 protein levels were increased following Ad-AMBRA1 transfected fibroblasts. Actin was used as a loading control. The AMBRA1/Actin densitometric ratios were marked (3 independent experiments gave similar results. See Figure S8). **b.** AMBRA1 up-expression reversed PUVA-SIPS and UVB-SIPS blocked the amount of Vps34 associated to BECLIN1 during autophagy. Protein extracts were immunoprecipitated (IP) using an anti-BECLIN1 antibody. Purified complexes and corresponding total extracts were analyzed by Western blotting with anti-Vps34 (left panels) and anti-BECLIN1 (right panels) antibodies. IP-Vps34/ total-Vps34 and IP-BECLIN1/ total-BECLIN1 ratios were marked (3 independent experiments gave similar results. See Figure S8). **c.** Prior to ultraviolet irradiation, cells were co-transfected with Ago-23a and Ad-AMBRA1 together with GFP-LC3 adenovirus, GFP-LC3 dot formation was assessed. Scale bars = 50μm. **d.** Quantitative analysis of GFP-LC3 positive cells in (c) (means ±SEM of the independent experiments, *n* = 3, N.S., not significant). **e.** Ago-23a reversed the Ad-AMBRA1-induced increase of LC3-I to LC3-II conversion and decrease of SQSTM1 in the fibroblasts (*n* = 3). Actin was used as a loading control. The LC3-II/LC3-I and SQSTM1/Actin ratios are marked (3 independent experiments gave similar results. See Figure S8).

**Figure 9:**
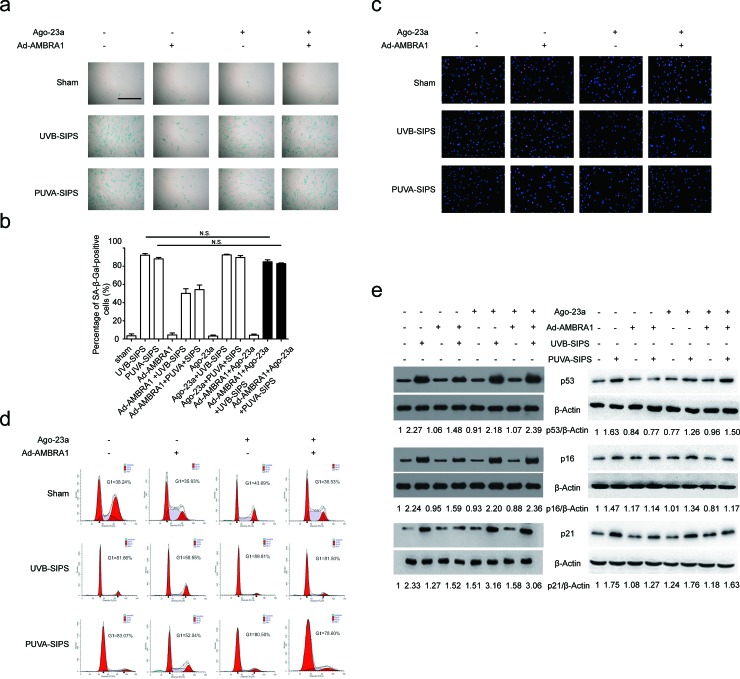
Overexpression of miR-23a blocked Ad-AMBRA1-induced anti-SIPS activity **a.** Prior to ultraviolet irradiation, cells were transfected with Ago-23a or control (Ago-CNT), SA-ß-gal staining was then performed with UV irradiation. Scale bars = 100μm. **b.** Quantitative analysis of SA-ß-gal-positive cells in (a) (means ±SEM of the independent experiments, *p < 0.05, N.S., not significant). **c.** The cells were stained by EdU and Hoechst two days post UV irradiation. The percentage of EdU-positive cells was shown **d.** The G1 arrest was examined in cells co-transfected with Ad-AMBRA1 and Ago-23a. (i) The relative protein levels of p53, p16, and p21 were examined. Actin was used as a loading control. The p53/Actin, p16/Actin and p21/Actin, densitometric ratios were marked (3 independent experiments gave similar results. See Figure S9).

After ultraviolet irradiation, the protein expression of AMBRA1 was significantly increased in the Ad-AMBRA1-transfected group compared with the Ad-CNT group (Figure 8a and Figure S8). Experiments consistently demonstrated that AMBRA1 up-regulation increased BECLIN1 interaction with associated kinase Vps34, as well as an increase in Vps34 activity in cells in which autophagy has been induced (Figure 8b and Figure S8). This finding suggests AMBRA1 has some regulatory effect overBECLIN1-Vps34 interaction. To confirm that AMBRA1 activated autophagic flux, similar autophagy tests were performed in cells transfected with either Ad-AMBRA1 or Ad-CNT. Indeed, transfection with Ad-AMBRA1 led to a prominent accumulation of GFP-LC3 dots and LC3-II protein and a significant decrease in SQSTM1 (Figure 8c-8e and Figure S8), suggesting normal autophagic flux with the upregulation of AMBRA1. However, these effects of Ad-AMBRA1were found to be suppressed in fibroblasts transfected by miR-23a agomirs (Figure 8c-8e and Figure S8). We also found that overexpression of AMBRA1 can result in an increase in the percentage of EdU-positive cells and a decrease in SA-*β*-gal positive cell percentages and G1 phase-arrested cell percentages, as well as an increase in p16, p53, and p21 protein levels in PUVA- and UVB-SIPS fibroblasts transfected by Ago-CNT (Figure 9a-9e and Figure S9). However, again these effects of AMBRA1were found to be suppressed in fibroblasts transfected by miR-23a agomirs.

## DISCUSSION

MiRNAs are implicated in a broad range of biological processes, including cell proliferation, differentiation, apoptosis, and stress response, linking them to numerous human diseases [21]. Additionally, recent studies have implicated novel roles of miRNAs in the regulation of the autophagy process [22, 23]. Our group previously found that miR-23a was up-regulated during UVB irradiation in mouse epidermis and keratinocytes, as well as human keratinocytes (cell line HaCaT) cells [13, 24, 25]. However, miR-23a has also been found to function as a growth-promoting and anti-apoptotic factor in hepatocellular carcinoma and gastric adenocarcinoma cell lines [26, 27]. We hypothesize that functional discrepancy is likely due to the difference in cellular conditions (senescence *versus* neoplasia).

Compared with classical transcriptional factors, miRNAs act mainly *via* regulation of their target genes at the mRNA level. For example, miR-23a induces cellular senescence by downregulating HAS2 expression and HA synthesis *in vitro* [14]. MiR-23a targets the LRP5 to inhibit osteogenic differentiation of human bone marrow-derived mesenchymal stem cells [28]. MiR-23a impairs bone differentiation in osteosarcoma *via* down-regulation of GJA1 [29]. In humans, miR-23a is predicted to be the most likely miRNA that regulates AMBRA1 (miRanda-mirSVR and Targetscan). AMBRA1 is a newly discovered autophagy-related gene (ATG), and its gene product, the AMBRA1 protein, is a crucial regulator of autophagy. Our experiments demonstratedmiR-23a binding sites on the 3′-UTR of AMBRA1 mRNA, and corresponding biological assays suggest that miR-23a suppresses AMBRA1 expression in humans. In SIPS, the expression of miR-23a was increased, whereas the autophagy levels and AMBRA1 expression were decreased. This indicates that miR-23a functions to repress the autophagy signaling pathway during the physiological development process. Depending on the physiological context and stress stimuli, autophagy can play a dual role: it can either enable escape from cell death or contribute to cell death (the latter is referred to as type II cell death or autophagic cell death). It has been recognized that the striking accumulation of autophagic vacuoles in cells likely reflects an imbalance between the rates of autophagic sequestration and completion of the degradation process. These cells can be thought of as undergoing ‘autophagic stress’. However, in our study, autophagy levels were significantly reduced in photoaged cells and the induction of autophagy by miR-23a inhibition or AMBRA1 induction only gained a relatively normal level of autophagy. Thus, it does not cause dysregulated autophagy which may contribute to excessive autophagic cell death and impaired function of fibroblasts.

By targeting AMBRA1, miR-23a affects several critical events in the autophagy pathway. The AMBRA1 protein interacts with Beclin1 through the target lipid kinase Vps34/PI3KC3 to assemble a class III PI3K complex, which positively regulates the formation of autophagosomes [30]. AMBRA1has been reported to bind to Beclin 1 and stabilizes the Beclin 1/Vps34 complex, thus promoting autophagosome formation. In this way, AMBRA1 acts as a crucial upstream regulator of autophagy initiation [31]. The interaction with Beclin 1 occurs along the central region of the AMBRA1protein (aa 533-780). Overexpression of this region has been shown to be sufficient to prompt autophagy [31]. Meanwhile this interaction with AMBRA1 occurs within a region on Beclin 1 adjacent to its BH3 domain, and is able to consistently compete with the binding of Bcl-2 to Beclin 1 [32]. The Beclin-1-Bcl-2 interaction has been linked to autophagy inhibition, and the relative amounts of Beclin 1 and Bcl-2 regulate the transition from cell homeostasis to apoptosis [33]. Thus, AMBRA1 is at the crossroads between autophagy and cell death [30]. The effects of the miR-23a-induced down-regulation of AMBRA1 in SIPS fibroblasts may indicate the inhibition of its autophagy while no obvious apoptosis is present. Our results show that AMBRA1 favors the BECLIN1-Vps34 functional interaction. Therefore, it is very likely that the binding sites between Beclin1 and AMBRA1are at the central region of the protein in our UV-irradiated model. Taken together, these results demonstrated that miR-23a-regulated autophagy mediates the development of SIPS.

While a number of aging-associated pathways have been characterized, the decrease in autophagic activity observed in almost all aging cells and tissues is believed to be a crucial contributor to the aging phenotype and aggravation of detrimental age-related diseases [15]. Furthermore, specific miRNAs are expressed differentially between normal and senescent cells. These miRNAs are potential targets for anti-photoaging therapies. In this study, miR-23a is a photo-sensitive miRNA that widely participates in the regulation of UV-induced photo-damage. While upregulation of miR-23a could accelerate the senescence of fibroblasts, Ant-23a-treated cells showed increased percentage of EdU-positive cells and decreased expressions of senescence-related proteins, G1 phase arrested cell percentage, and SA-β-gal positive cell percentages. Treatment with both Rapamycin (an mTOR inhibitor) and AMBRA1 both had an anti-SIPS effects and dramatically promoted autophagy. However, these effects could also be mitigated by treatment with Ago-23a. In conclusion, mir-23a plays an important role in regulation of aging by inhibiting AMBRA1 expression and autophagy.

As previously discussed, miR-23a, miR-24, and miR-27a are in the same gene cluster, but up-regulation of miR-27a and miR-24 does not produce the same effects as miR-23a. One explanation for this discrepancy is that miR-23a is closest to transcriptional factor binding sites. Surprisingly, down-regulated miR-23a, but not miR-27a or miR-24, is necessary for reducing the SA-β-gal percentage and increasing EdU-positive cell percentage, which is the hallmark of PUVA-SIPS and UVB-SIPS fibroblasts. Thus, it is apparent that miR-23a initiates senescence following ultraviolet irradiation, whereas miR-27a and miR-24 do not exert a synergistic effect. On the other hand, a dozen miRNAs including miR-27a and miR-24 have been shown to be up-regulated in skin cells upon ultraviolet irradiation [25], and miR-24 overexpression can induce enhanced autophagy in smooth muscle cells [34]. Therefore, further studies are required to specifically elucidate the role of miR-27a and miR-24 in photoaging.

The regulation of autophagy by miRNAs may offer an efficient multitarget strategy to accomplish modest autophagy inhibition. Future *in vivo* studies should be conducted to explore the balance between the potential beneficial effects and side effects of autophagy modulation by miR-23a, antagomirs, and their derivatives. Finally, studies on the expression levels of miR-23a in diseases with autophagy abnormalities may reveal the potential of this miRNA as a disease marker.

## MATERIALS AND METHODS

### Cell culture

Normal human skin samples were obtained from circumcisions in accordance with the ethical committee approval process of Jiangsu Provincial People's Hospital, Nanjing, Jiangsu, China. The study was approved by the Local Ethics Committees of the First Affiliated Hospital with Nanjing Medical University, Nanjing, Jiangsu, China. Written informed consent was obtained from all participants in this study. Specimens were sterilized in 70% ethanol, minced, and incubated in Dulbecco's modified Eagle medium (DMEM) supplemented with 10% fetal bovine serum and 1% penicillin-streptomycin in an atmosphere of 5% CO_2_ at 37°C. Dermal HDFs normally grew from the explants after 5-7 days. The cells from passages 3 to 6 were used in this study.

### Establishment of *in vitro* UV-SIPS models

UVB-SIPS and PUVA-SIPS fibroblast models were established according to previous reports. For the UVB-SIPS model, the dosage of UVB was introduced to the cells according to pilot experiments. UVB irradiation was delivered with a Philips TL 20W/12 (Eindhoven, The Netherlands), which is a fluorescent bulb emitting 280-320 nm wavelengths with a peak of 313 nm. The UVB-SIPS induction was processed using previously described methods. Before UVB irradiation, the medium was removed and covered with phosphate buffered saline (PBS). Irradiation output was monitored with a Waldmann UV meter (Waldmann, Villigen-Schwenningen, Germany). 25 mJ/cm^2^ UVB was performed twice per day for 5 days. At 48 hours after the last stress, the cells were harvest for further detection.

For the PUVA-SIPS model, the UVA irradiation was conducted using a UVA light source (Philips) with a UVB blocking filter. The dosage of UVA irradiation was determined using an IL-1700 radiometer (International Light) equipped with a UVA detector (326-401 nm). The cells were irradiated at a distance of 15 cm. The PUVA-SIPS induction was processed using previously described methods [3]. The fibroblasts were pre-incubated with 8-methoxypsoralen (100 ng/ml) for 24 hours and subsequently received UVA irradiation at a dosage of 9 J/cm^2^. At 7 days after the UVA stress, the cells were harvested for further evaluation.

### SA-ß-gal staining for the detection of senescent cells

To measure one of the biomarkers of senescence, senescence-associated ß-galactosidase (SA-ß-gal) staining was performed. Cells were fixed in 2% formaldehyde/0.2% glutaraldehyde, rinsed with PBS, and incubated at 37°C with fresh SA-ß-gal stain solution, which is composed of 1 mg of 5-bromo-4-chloro-3-indolyl ß-D galactoside per mL (stock = 20 mg of dimethylformamide per mL), 40 mM citric acid, sodium phosphate (pH 6.0), 5 mM potassium ferrocyanide, 150 mM NaCl, and 2 mM MgCl_2_.

### Proliferation assay

Each group of fibroblasts was incubated for an additional 2 h in respective medium containing 50 μM EdU (RiboBio, Guangzhou, China). Cells were then washed with PBS, fixed and permeabilized with PBS containing 4% paraformaldehyde and 0.5% triton X-100. Cells were incubated with 1× Apollo reaction cocktail (100 μl/well) for 30 min. DNA was incubated with Hoechst 33342 stain (100 μl/well) for 30 min. For each EdU experiment, five random fields were imaged by 100× magnification. Captured images were processed and analyzed with ImageJ software. The number of EdU positive cells was identified by Hoechst nuclei staining and expressed as a percentage of the total number of cells in each field

### Recombinant adenovirus vector for AMBRA1 overexpression

The recombinant adenovirus vector for AMBRA1 (Ad-AMBRA1) and a negative adenovirus vector (Ad-CNT) were produced by Shanghai GeneChem Co. Ltd (Shanghai, China). The vectors included a GFP sequence, which served as a marker gene. A high-titer adenovirus stock was made after several rounds of amplification in the 293T cells. Western blotting confirmed transgenic expression of the recombinant adenoviruses in our fibroblast models. The fibroblasts were infected with Ad-AMBRA1 or a negative vector at a 10x multiplicity of infection for 48 h and then subjected to experiments after being deprived of serum for 12 h.

### Adenoviruses for GFP-LC3

The tandem fluorescent GFP-LC3 (Ad-tf-LC3) (purchased from HanbioCo. Ltd, Shanghai, China) was used to generate an adenovirus (Ad-tf-LC3). Fibroblasts were transduced with 15 multiplicities of infection (MOIs) of adenovirus for 24 h.

### Evaluation of fluorescent LC3 puncta

Fibroblasts cultured on coverslips were transduced with Ad-tf-LC3 at 15 MOI. Twenty-four hours after adenovirus transduction, the cells were washed with PBS, fixed with 4% paraformaldehyde for 20 min, mounted with a reagent containing, and viewed with confocal microscopy. The number of GFP dots was determined by manually counting fluorescent puncta in five fields from three different myocyte preparations. The number of dots per cell was obtained by dividing the total number of dots by the number of nuclei in each microscopic field. At least 150 GFP positive cells per condition were counted and the graphs were plotted as percentage of GFP-LC3 dot positive cells over total number of transfected cells.

### Bioinformatic analysis of miR-23a target genes

The putative miR-23a targets were predicted using several different algorithms, including TargetScan (http://www.targetscan.org/), PicTar (http://pictar.bio.nyu.edu/) and miRanda (http://microrna.sanger.ac.uk/). An interaction between miR-23a and the 3′-UTR of its target gene was predicted using RNAhybrid (http://bibiserv.techfak. uni-bielefeld.de/rnahybrid/).

### Construction of 3′-UTR reporter plasmids and luciferase assays

The 3′-UTR segments of AMBRA1 containing the miR-23a binding sites were amplified *via* PCR from the genomic DNA of 293T cells (AMBRA1-F: 5′-CCGCTCGAGAGACAAACGTTGCACTGGTGC-3′, AMBRA1-R: 5′-GAATGCGGCCGCGCGAGGGGCATGTCATCAT-3′) and were cloned into the XhoI and NotI sites downstream of the luciferase reporter gene of the pmiR-RB-Report^TM^ vector (RiboBio), which was named pmiR-AMBRA1-3′-UTR-wt. Mutations in the predicted miR-23a binding sites were generated using the KOD -Plus- Mutagenesis Kit (TOYOBO, Osaka, Japan) with pmiR-AMBRA1-3′-UTR-wt as a template. This was named pmiR-AMBRA1-3′-UTR-mut. All constructs were verified *via* DNA sequencing. The 293T cells were co-transfected in 96-well plates with either miR-23a mimics or a negative control and either pmiR-AMBRA1-3′-UTR-wt or pmiR-AMBRA1-3′-UTR-mut *via* Lipofectamine 2000. After 36 h of transfection, the luciferase activity was measured using the dual luciferase assay system (Promega, Heidelberg, Germany). The firefly luciferase activity of each sample was normalized to the Renilla luciferase activity.

### Statistical analysis

All experiments were repeated at least three times and representative experiments are shown. Data are presented as means ± SEM. Differences were evaluated by one-way analysis of variance *post hoc* Dun net's, using computer program GraphPad Prism (GraphPad Software, San Diego, CA). A *p*-value of less than 0.05 was considered statistically significant.

## SUPPLEMENTARY MATERIALS AND METHODS, FIGURES


