# Supplementary material for: MiR-23a-depressed autophagy is a participant in PUVA- and UVB-induced premature senescence
Source: Oncotarget. 2016 May 13;7(25):37420–35. doi: 10.18632/oncotarget.9357 (PMC5122322; doi:10.18632/oncotarget.9357)
Supplement: Supplementary file 1 [file oncotarget-07-37420-s001.pdf]

MiR-23a-depressed autophagy is a participant in PUVA- and UVB-induced premature senescence

Supplementary Materials and Methods

MicroRNA agomir or antagomir transfection

For inhibition of miR-23a, miR-27a, miR-24, a specific inhibitor, Antagomir(Ribobio Co., Guangzhou, China) was used.Antagomir Negative Control was used as a control.

To establish an overexpression of miR-23a, Agomir (Ribobio Co., Guangzhou, China), thio-, cholesterol-modified miRNA mimic (double-stranded RNAs) was used to transfect fibroblasts, and Agomir-CNT, which is similar to Agomir-23a but with a scramble seeding sequenceused as control. Briefly, cells in wells of 96-well/6-well plates were grown to 50–70% confluence. Either AgomirorAntagomir was transfected into the cells at a concentration of 100 nM in the presence of Lipofectin Reagent (Invitrogen) according to the manufacturer's protocol.

qRT-PCR analysis for miRNA and mRNA expression level

The total RNA isolation and miRNA enrichment were performed with the mirVana miRNA Isolation Kit (Ambion, Austin, TX, USA) according to the manufacturer's instructions. The RNA concentration was quantified with a NanoDrop spectrophotometer (Thermo Fisher, Waltham, MA, USA). Small RNA enrichment was performed using a mirVana miRNA Isolation Kit (Ambion, Austin, TX, USA) and quantified using the mirVana™ qRT-PCR miRNA Detection Kit (Ambion, Austin, TX, USA). For real-time PCR, SYBR Green I was used for the quantification of miRNA transcripts following the manufacturer's instructions. The appropriate cycle threshold (Ct) was

determined using the automatic baseline determination feature. Reactions containing qRT-PCR primer sets were specific for human miR-23a (F: 5`-ACACTCCAGCTGGGATCACATTGCCAGGG-3`, R: 50-CTCAACTGGTGTCGTGGAGTCGGCAATTCAGTTGAGGGAAATCC-30), miR-24 (F: 5`-ACACTCCAGCTGGGTGGCTCAGTTCAGCAG-3`, R: 5`-CTCAACTGGTGTCGTGGAGTCGGCAATTCAGTTGAGCTGTTCCT-3`) miR-27a (F: 5`-ACACTCCAGCTGGGTTCACAGTGGCTAAG-3`, R: 5`-CTCAACTGGTGTCGTGGAGTCGGCAATTCAGTTGAGGCGGAACT-3`), AMBRA1 (F: 5`-AACCCTCCACTGCGAGTTGA-3`, R: 5`-TCTACCTGTTCCGTGGTTCTCC-3`). As an internal control, U6 (F: 5`-ATTGGAACGATACAGAGAAGATT-3`, R: 5`-GGAACGCTTCACGAATTTG-3`) was used for miRNA template normalization, GAPDH (F: 5`-CCTCATCCACCCCATCCCT-3`, R: 5`-GTCCATAGCCTCTACTGCCA-3`)

Flow cytometry for the detection of cells in phase G1

To determine whether the UVB-stressed HDFs exhibit cell growth arrest, a cell cycle analysis with flow cytometry was performed. The HDFs were fixed with 70% alcohol, washed twice with PBS, digested with RNase and stained with propidium iodide. A flow cytometer (FAC-Scan, BD Biosciences, San Diego, CA, USA) was used to gather data and images, to analyze the cell cycle, and to calculate the percentage of cells in the G1 phase.

Immunoprecipitation assay

In the immunoprecipitation experiments, the cells were lysed in HEMG buffer (25 mM HEPES (pH 8.0), 100 mM NaCl, 0.5% Nonidet P-40, 0.1 mM EDTA, 10% glycerol) plus protease and phosphatase inhibitors (protease inhibitor cocktail, 1 mM sodium fluoride, 1 mM sodium orthovanadate, 1 mM sodium molibdate; Sigma-Aldrich). The lysates (1–2 mg) were then incubated with rotation at 4 °C for 30 min. After a centrifugation at 4 °C for 10 min at 13,000 g to remove the insoluble debris, equal amounts of protein were incubated with 2 mg of Beclin1 antibody (BD Biosciences, San Diego, CA, USA) overnight at 4 °C, followed by 60 min incubation with 20 ml of protein A/G sepharose beads (Amersham Bioscience Corp, Piscataway, NJ). The beads were finally collected via centrifugation and washed four times with the HEMG buffer. The proteins that were bound to the beads were eluted with 50 ml of SDS–polyacrylamide gel electrophoresis sample buffer, heated to 70 °C for 10 min and then subjected to western blotting.

Western Blotting assay

The cells were lysed in 62.5 mM Tris–HCl (pH 6.8) containing 2% w/v SDS, and the protein concentration was determined using the Pierce BCA assay (Thermo Fisher Scientific, Rockford, IL, USA). Mercaptoethanol and bromophenol blue were added to make the final composition equivalent to the Laemmli sample buffer. The samples were fractionated using SDS-polyacrylamide gel electrophoresis (SDS-PAGE) and blotted onto Immobilon-P membrane (Millipore, Billerica, MA, USA). Rabbit anti-mouse HRP (1:1,000 dilution) and goat anti-rabbit

HRP (1:1,000 dilution) were used as secondary antibodies (Biotime, Haimen, China). Antibody binding was visualized via Pierce ECL reagents (Thermo Fisher Scientific, CA, USA). We used mouse monoclonal antibodies against p53, $p21^{WAF-1}$ and rabbit polyclonal anti-Beclin1 (1:500 dilution, Cell Signaling Technology, California, USA), rabbit monoclonal antibody against $p16^{INK-4a}$ (1:500 dilution, Bethyl Laboratories, Montgomery, TX, USA), and rabbit polyclonal against SQSTM1/p62 (1:1,000 dilution, BD Transduction Laboratories, San Diego, CA, USA). We used rabbit anti-AMBRA1 (1:1,000; Strategic Diagnostic Inc., Cambridge, UK), rabbit polyclonal anti-Vps34 (1:500 dilution, Invitrogen, Carlsbad, CA, USA), rabbit polyclonal anti-LC3 (1:250 dilution, MBL, Japan) and monoclonal anti-β-actin antibody (1:1,000 dilution, Biotime, Haimen, China) as controls. The quantification of protein bands was established using Band-Scan software (PROZYME, San Leandro®, California).

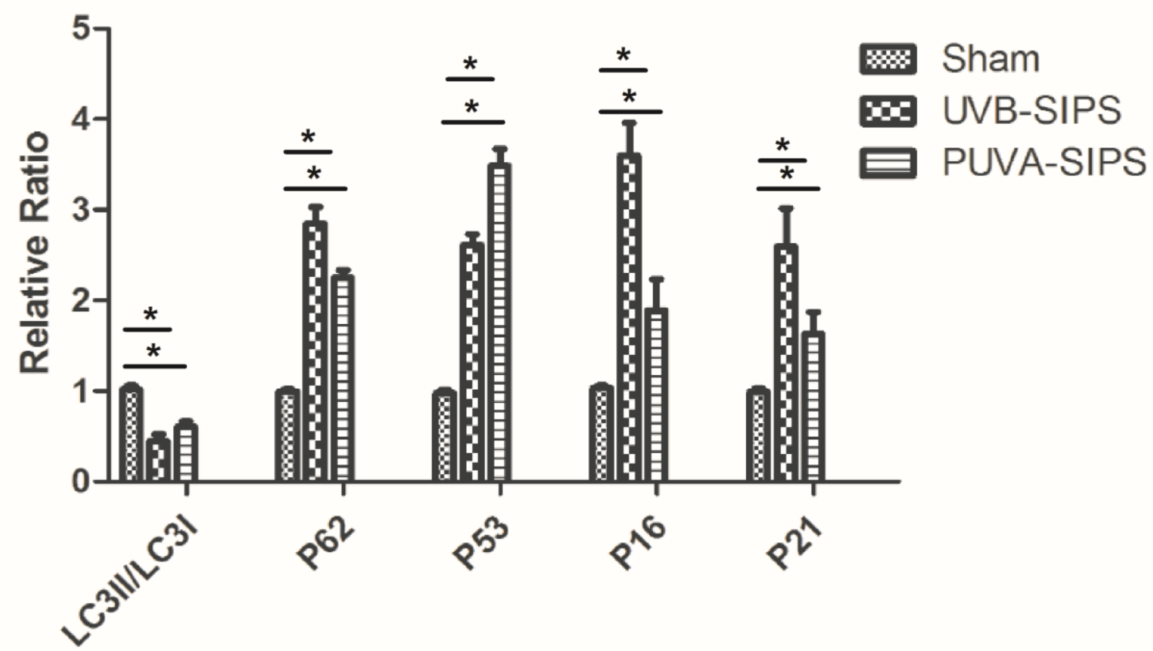

Figure S1

Blots from triplicate experiments were scanned, and densitometric values corrected for actin were plotted as mean±SEM. *P < 0.05.

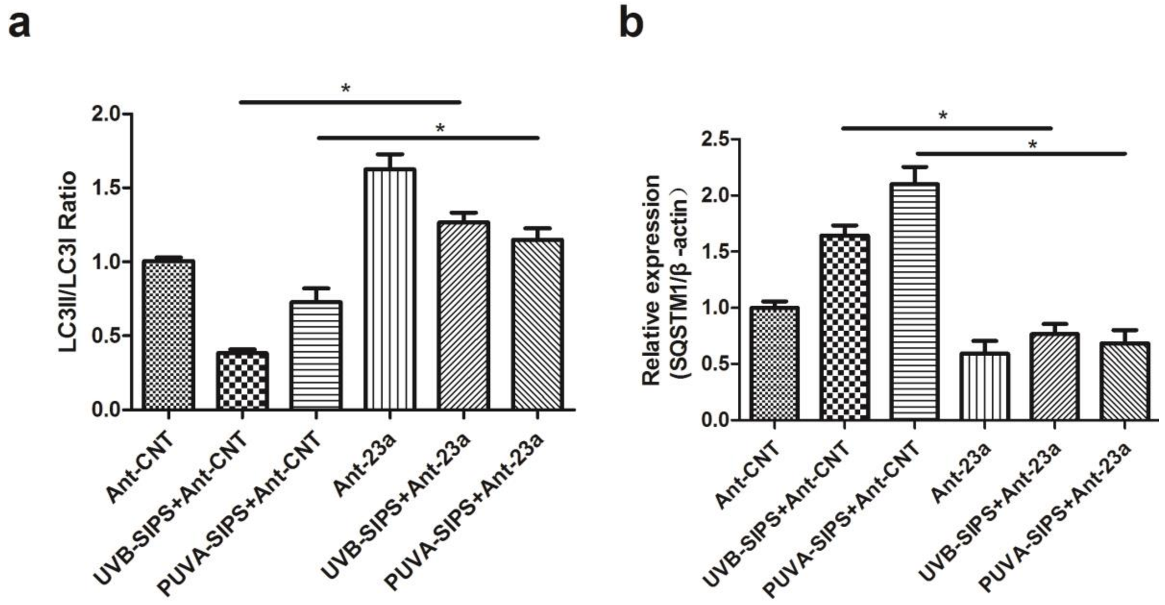

Figure S2

Blots from triplicate experiments were scanned, and densitometric values corrected for actin were plotted as mean±SEM. *P < 0.05.

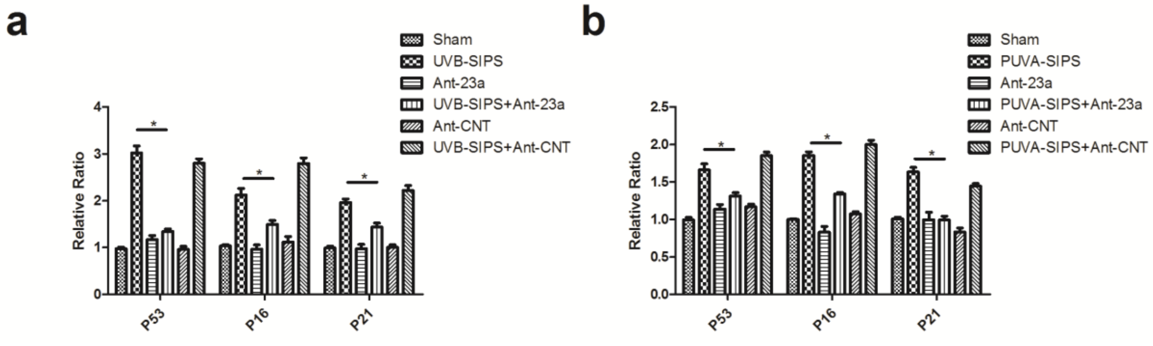

Figure S3

Blots from triplicate experiments were scanned, and densitometric values corrected for actin were plotted as mean±SEM. *P < 0.05.

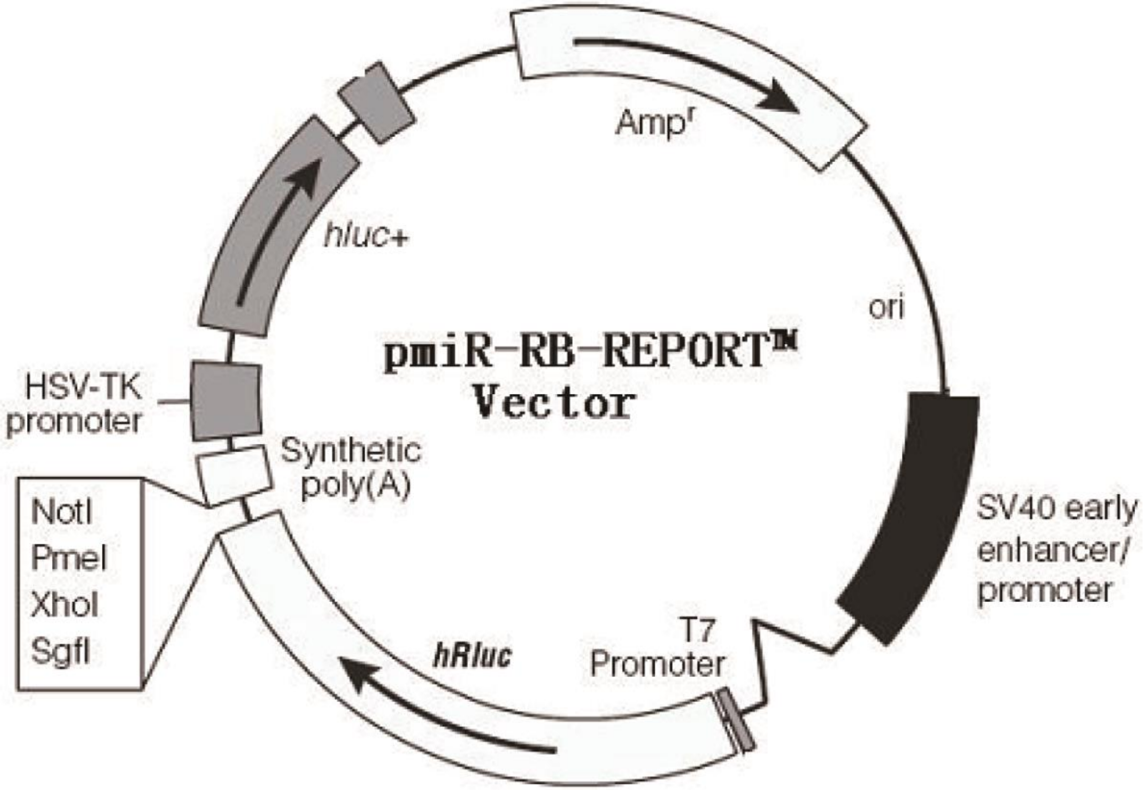

Figure S4

Schematic diagram of luciferase reporter vector pmiR-RB-Report™. Putative miR-23a regulatory elements predicted via bioinformatics were cloned into the 3`-untranslated region of the luciferase gene at the XhoI and NotI sites (*$p < 0.05$).

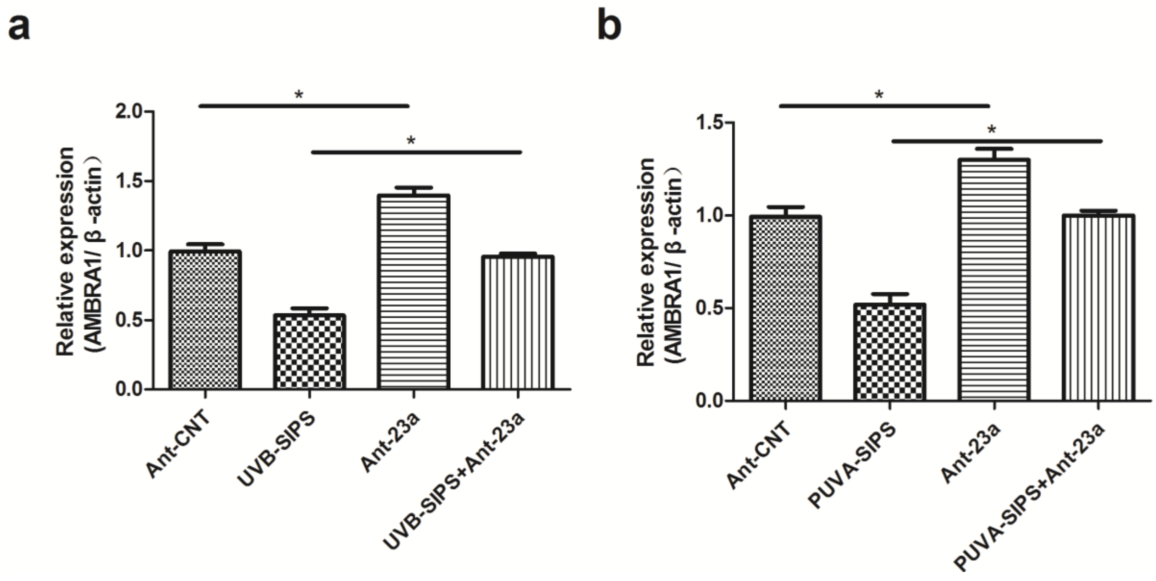

Figure S5

Blots from triplicate experiments were scanned, and densitometric values corrected for actin were plotted as mean±SEM. *P < 0.05.

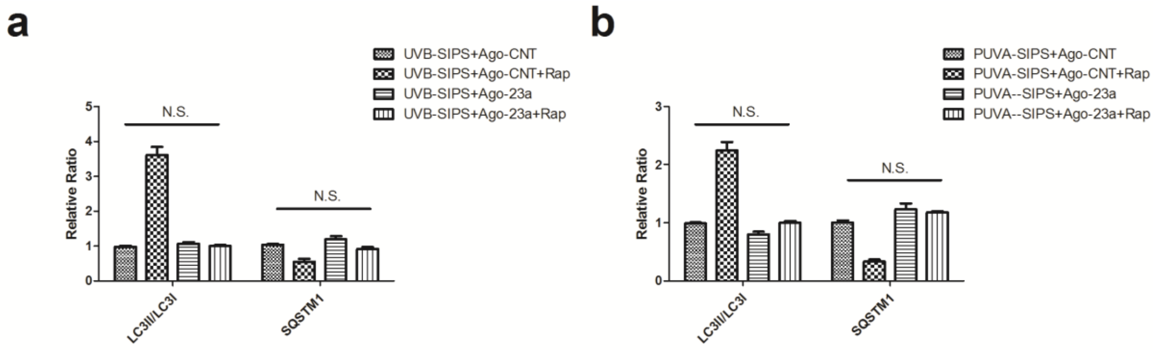

Figure S6

Blots from triplicate experiments were scanned, and densitometric values corrected for actin were plotted as mean±SEM. *P < 0.05.

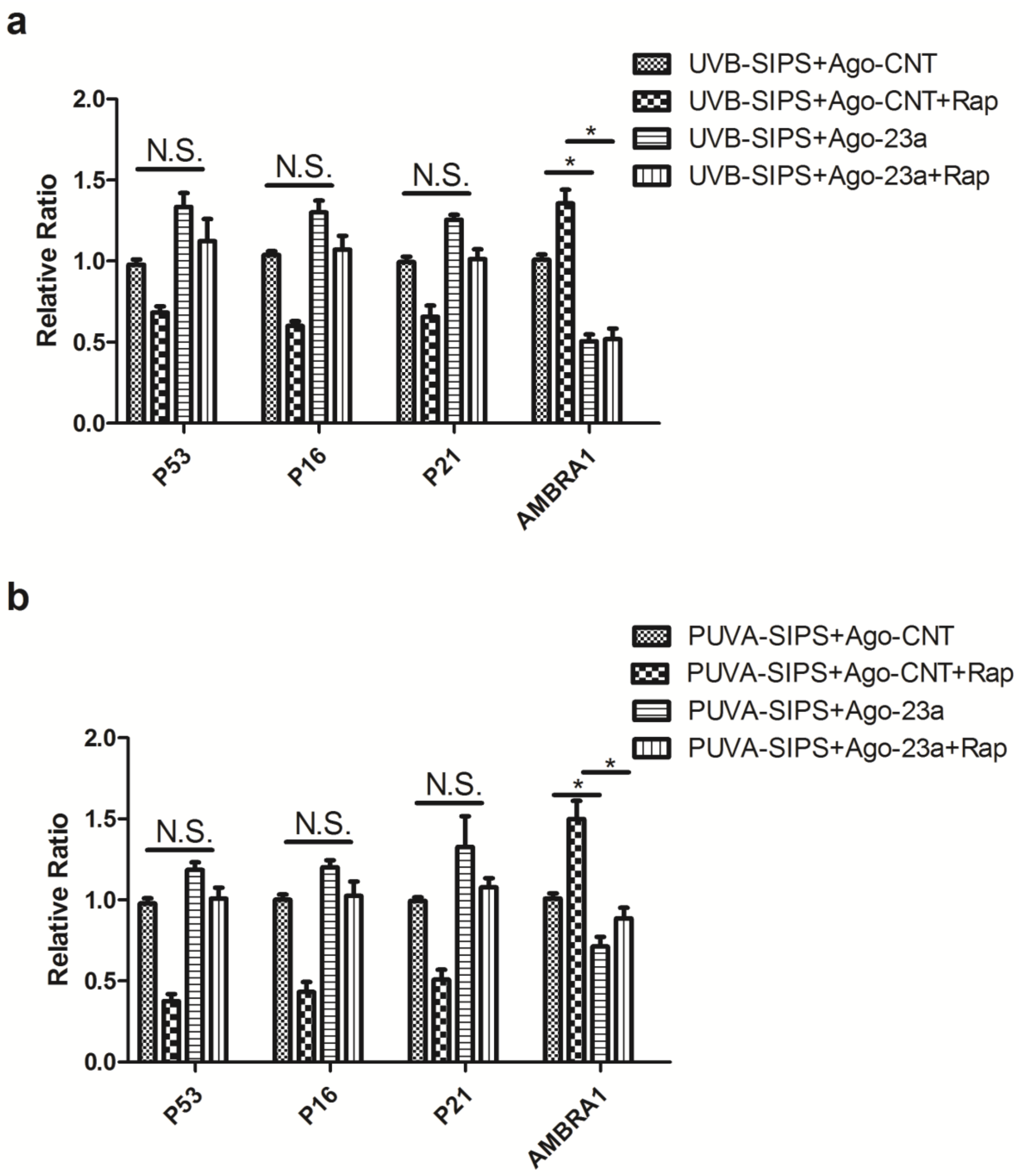

Figure S7

Blots from triplicate experiments were scanned, and densitometric values corrected for actin were plotted as mean±SEM. *$P < 0.05$.

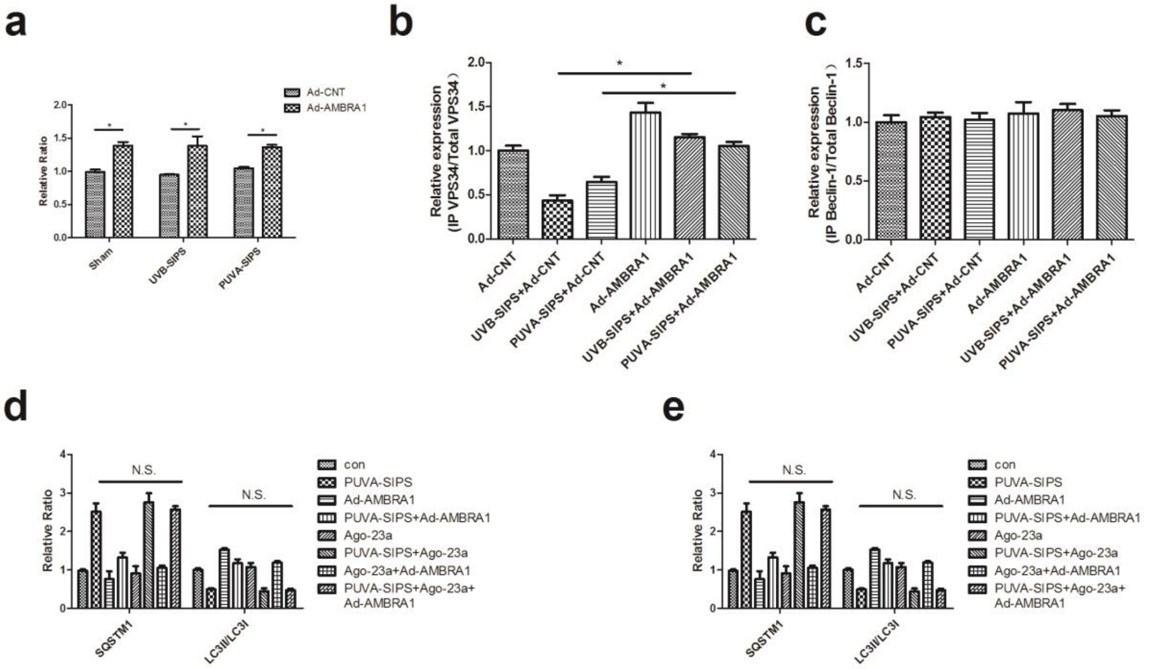

Figure S8

Blots from triplicate experiments were scanned, and densitometric values corrected for actin were plotted as mean±SEM. *$P < 0.05$.

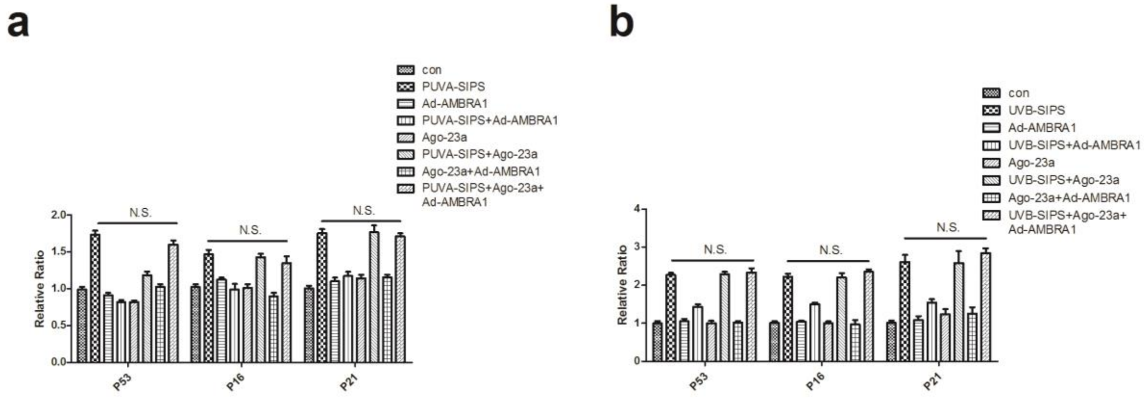

Figure S9

Blots from triplicate experiments were scanned, and densitometric values corrected for actin were plotted as mean±SEM. *P < 0.05.
